# Indirect composite restorations luted with two different procedures: A ten years follow up clinical trial

**DOI:** 10.4317/jced.51604

**Published:** 2015-02-01

**Authors:** Nicola Barabanti, Alessandro Preti, Michele Vano, Giacomo Derchi, Francesco Mangani, Antonio Cerutti

**Affiliations:** 1 Assistant Professor. Department of Restorative Dentistry, University of Bre-scia, Brescia, Italy; 2 Assistant Professor. Department of Surgical Pathology, Medicine, Molecular and Critical Area, University of Pisa, Pisa, Italy; 3 Research Fellow. Department of Surgical Pathology, Medicine, Molecular and Critical Area, University of Pisa, Pisa, Italy; 4 Head Professor Operative and Esthetic Dentistry. Department Università Tor Vergata, Rome, Italy; 5 Head Professor. Department of Restorative Dentistry, University of Brescia, Brescia, Italy

## Abstract

Objectives: The aim of this clinical trial was to evaluate posterior indirect composite resin restoration ten years after placement luted with two different procedures. Study Design: In 23 patients 22 inlays/onlays (Group A) were luted using a dual-cured resin composite cement and 26 inlays/onlays (Group B) were luted using a light cured resin composite for a total of 48 Class I and Class II indirect composite resin inlays and onlays. The restorations were evaluated at 2 time points: 1) one week after placement (baseline evaluation) and 2) ten years after placement using the modified USPHS criteria. The Mann-Whitney and the Wilcoxon tests were used to examine the difference between the results of the baseline and 10 years evaluation for each criteria. Results: Numerical but not statistically significant differences were noted on any of the recorded clinical parameters (p>0.05) between the inlay/onlays of Group A and Group B. 91% and 94 % of Group A and B respectively were rated as clinically acceptable in all the evaluated criteria ten years after clinical function. Conclusions: Within the limits of the study the results showed after ten years of function a comparable clinical performance of indirect composite resin inlays/onlays placed with a light cure or dual cure luting procedures.

** Key words:**Light curing composite, dual curing composite, indirect composite restoration, inlays/onlays, clinical trial.

## Introduction

Dental clinician has to face everyday difficult tasks when restoring posterior teeth, particularly in large cavity, where he has to decide which material and technique is more adequate for the restoration. Nowadays esthetic considerations play a major role in the treatment planning of dental care. Therefore amalgam and gold restoration, even though showed good long term results are no more accepted by patients (1). Esthetic alternatives include direct composites, composite inlays/onlays, and ceramic inlays/onlays.

Direct composite restorations for posterior teeth are preferred by many clinicians for reasons of minimal intervention (2,3). They are made in one treatment session at relatively low costs. However in posterior cavities, especially with the cervical margin situated in dentin, the mass to be polymerised is so large that the shrinkage forces prevail, thereby producing marginal gaps and defects (4). This promotes micro-leakage, which can lead to secondary caries, pulp irritation, postoperative sensitivity and marginal discolouration.

Therefore direct composite resin restorations for the rehabilitation of severely damaged or fractured posterior teeth may be inadequate in the long term due to insufficient wear resistance, imperfect proximal or occlusal morphology and deficient mechanical properties (5). In order to overcome these problems indirect techniques were introduced. According to the definition, inlays are single-tooth restorations that compensate a proximal-occlusal or gingival lesion with minimal or moderate extensions, whereas onlays cover the occlusal surface with a wide mesio-occluso-distal restoration (6). For such restorations both indirect composite resin and ceramic showed good results however composite resin has the advantage to be less expensive and more user-friendly and repairable than ceramic (7).

Laboratory-processed composite inlays/onlays are more resistant to occlusal wear than direct composites, particularly in occlusal contact areas and show a reduced polymerization shrinkage which is limited to the thin luting layer (8-10). Therefore are usually indicated for the restoration of large defects (11).

Several clinical studies showed high success rate for indirect composite inlays in short and medium term follow up. In particular a succes rate of 90% (12) and 97.5% (13) respectively after two and five years was reported for indirect composite inlays. However little information is available in the literature on the long-term succes rate of indirect composite restoration.

Tooth-colored inlays and on lays are routinely bonded to the tooth substrate employing dual-curing or light-curing cements (14,15). Dual-curing materials are advantaged by their self-curing component, which favours the conversion even in presence of scarce radiant energy, but have the disadvantage of requiring the mixture of two elements [responsible for the formation of porosities or voids and for the incorporation of bubbles] and skillful handling being considerably fluid.

On the other hand light-curing composites are easily handled and are characterised by controllable hardening times that create high quality margins, but their light only activation constitutes a disadvantage (16). Few clinical studies analysed the use of solely light-curing composite resins as a bonding material producing conflicting results (17).

To our knowledge there is a paucity of clinical information regarding which luting procedure is more adequate to bond indirect composite restoration to dentin.

Among the evaluation systems to assess the performance of restorations the most commonly used is the modified US Public Health Service [USPHS] evaluation system. This system was designed to measure clinically important features of dental restorations (18) such as color match, marginal discoloration, anatomic form, marginal adaptation, and caries.

In 2007 Hickel *et al.* introduced a new proposal for clinical testing protocol for controlled clinical trials, however the present study started earlier and therefore is still based on modified USPHS criteria (19,20).

The aim of this clinical trial was to evaluate posterior indirect composite resin restoration ten years after placement using the modified USPHS scoring system. The following null hypotheses were tested:

1. The clinical performance of indirect composite resin restoration cemented using two different luting systems did not exhibit significantly different results.

2. The clinical performance of indirect composite resin restoration is reliable for the restoration of large defects on long term follow up.

## Material and Methods

With approval from the Ethics Committee of the University of Brescia, 28 young adult patients were selected from a pool of candidates that included routine polyclinic patients of the dental school clinic. Written informed consent forms were obtained from all patients at the start of this research study. The clinical procedures of cavity preparation and restoration placement were performed by one experienced dentist from the Department of Restorative Dentistry at Brescia University Hospital.

The indications for placement of the indirect composite restorations were large, multi-surfaced cavities on permanent upper and lower premolars and molars involving at least one cusp. All restorations included for evaluation in this study had all-enamel margins, were in occlusion at baseline, and had no pulp exposure at placement. In addition the following requirements had to be met in a tooth which was to be restored with a composite inlay or onlay: 1] absence of pain 2] absence of pathological changes in the periapical region 3] opportunity to apply rubber dam during the placement procedure of an inlay or onlay.

Data presented in this report were derived from indirect composite restorations placed over a period of two years [January 2000–January 2002] and evaluated ten years after placement.

28 patients were initially enrolled in this study. 5 patients were then excluded because they were not meeting the inclusion criteria. In 23 patients on a randomized basis 22 inlays/onlays [Group A] were luted using a dual-cured resin composite cement [Calibra, Dentsply, Woodbridge, Ontario, Canada] and 26 inlays/onlays [Group B] were luted using a light cured resin composite [Filtex Z250, 3M ESPE, St. Paul, MN, USA] for a total of 48 Class I and Class II indirect composite resin inlays and onlays. The compositions of the materials used for luting the indirect restorations are summarized in  Table 1.

**Table 1:**
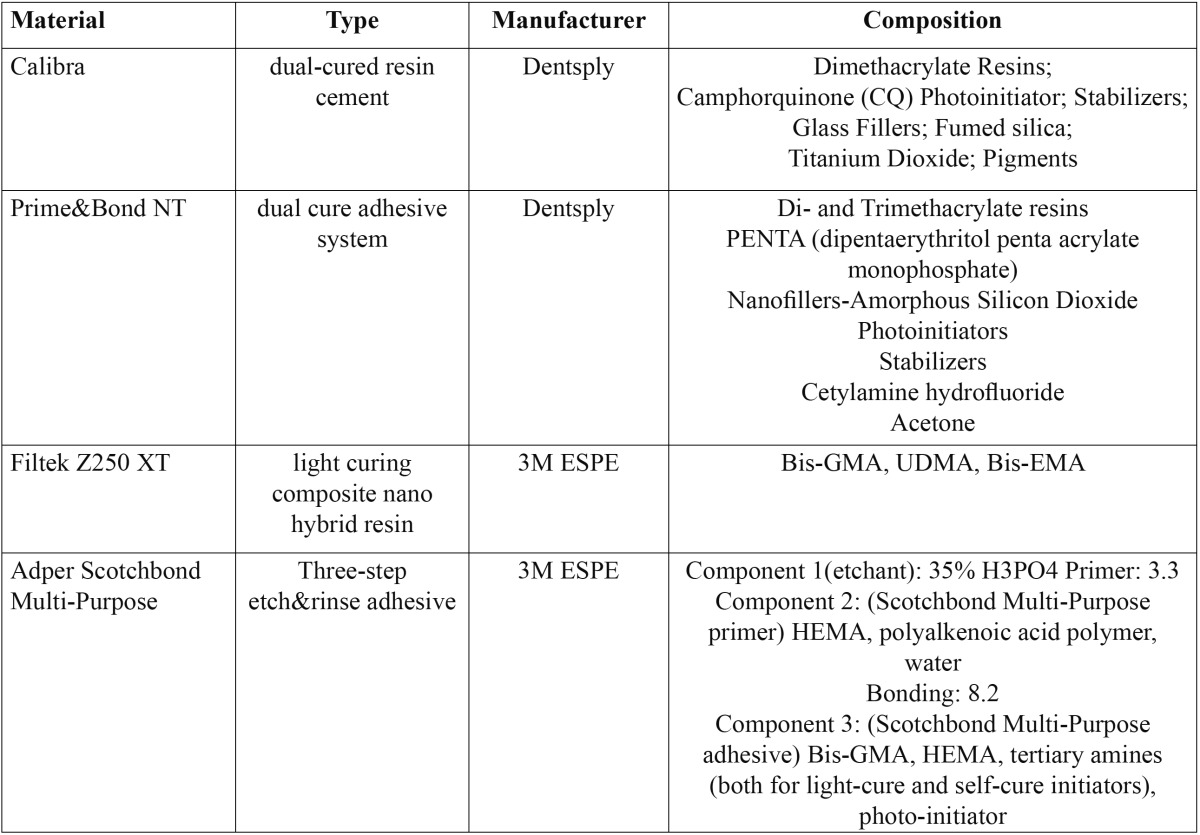
Materials manufacturer and compositions as compiled from manufacturers data.

All teeth were in occlusion and had at least one proximal contact with an adjacent tooth.

-Clinical Procedure for Indirect Composite Inlays/onlays:

All cavities were prepared according to the common principles for adhesive inlays and onlays. To achieve convergence angles between opposing walls at an estimated 10°-12, cavities were prepared with 80-lm–grit diamond burs and finished with 25-lm–grit diamond burs under water cooling. Care was taken to minimize increases in cavity extension. The cavities were prepared with rounded inner line angles and to a depth that allowed for at least 2 mm of resin material at the occlusal contact area. All undercuts were eliminated. Complete arch impressions were taken with a polyether material [Impregum F, Espe] followed by disinfecting the impressions for 10 min [Impresept, Espe] and rinsing them under running water for 15 s. Provisional restorations [Clip, Voco] were placed with eugenol-free temporary cement [Provicol, Voco, Cuxhaven, Germany]. All inlays were made by a dental technician who was experienced in fabricating composite resin inlays strictly following manufacturer’s instructions. The inlays were postcured in a light-oven [Uni-XS, Kulzer, Wehrheim, Germany] for 10 min to improve the physical properties. One laboratory technician prepared all the inlays and onlays following the manufacturers’ instructions. All inlays and onlays were definitively inserted within 1 weeks after impression. After removal of provisional restorations, the teeth were thoroughly cleaned with a prophylaxis brush and pumice. Rubber dam was used in all cases. After try-in of the inlays to check proximal contacts and marginal fit, all adhesive surfaces of the inlays were sandblasted [Al2O3 50 mm, 2 bar], subsequently cleaned with ethanol, and air-dried. A silane coupling agent [Monodond S, Vivadent] was applied to all internal inlay surfaces. Enamel margins were etched using phosphoric acid [Etching Gel Siringe, 3M ESPE, St. Paul, MN, USA] for 30 s and dentine for 15 s, followed by thorough washing of all surfaces with water and subsequent drying of the prepar-tions with oil-free compressed air. Care was taken to avoid desiccation of the tooth substrate. The indirect composite restorations were cemented according to the two study groups:

Group A, a dual-curing resin cement was used [Calibra, Dentsply, Woodbridge, Ontario, Canada] with a dual cure adhesive system [Prime&Bond NT, Dentsply, DeTrey, Weybridge Germany];

Group B, a light curing composite nano hybrid resin was used [Filtek Z250, 3M ESPE, St. Paul, MN, USA] with a three-step etch&rinse adhesive system [Scothbond Multiporpouse 3M ESPE, St. Paul, MN, USA].

Excess resin cement was removed in all cases with an explorer, a brush and dental floss interproximally. Each inlay surface was light-cured for 40 s with a polymerization light [Elipar Highlight, 3M Espe, Seefeld, Germany]. After placement and removal of rubber dam, static and dynamic occlusion was adjusted using fine grit dia-mond burs, then inlays were finished with disks and strips [Sof-Lex, 3M Dental Products, St. Paul, MN, USA].

-Clinical Evaluation

Restorations were rated blindly by one experienced and calibrated dentists who was not involved with the inser-tion of the indirect composite inlays/onlays and who did not know which materials were used to lute the inlay/onlays on the teeth that he was evaluating. Restorations were assessed with a mirror and probe. In addition digital intraoral photographs and intraoral radiograph were taken. The restorations were evaluated at 2 time points: 1] one week after placement [baseline evaluation] and 2] ten years after placement using the modified USPHS criteria ( Table 2). This clinical assessment method resulted in ordinally structured data for the outcome variables [Alpha = excellent result; Bravo = acceptable result; Charlie = unacceptable, replacement of the restoration necessary].

**Table 2:**
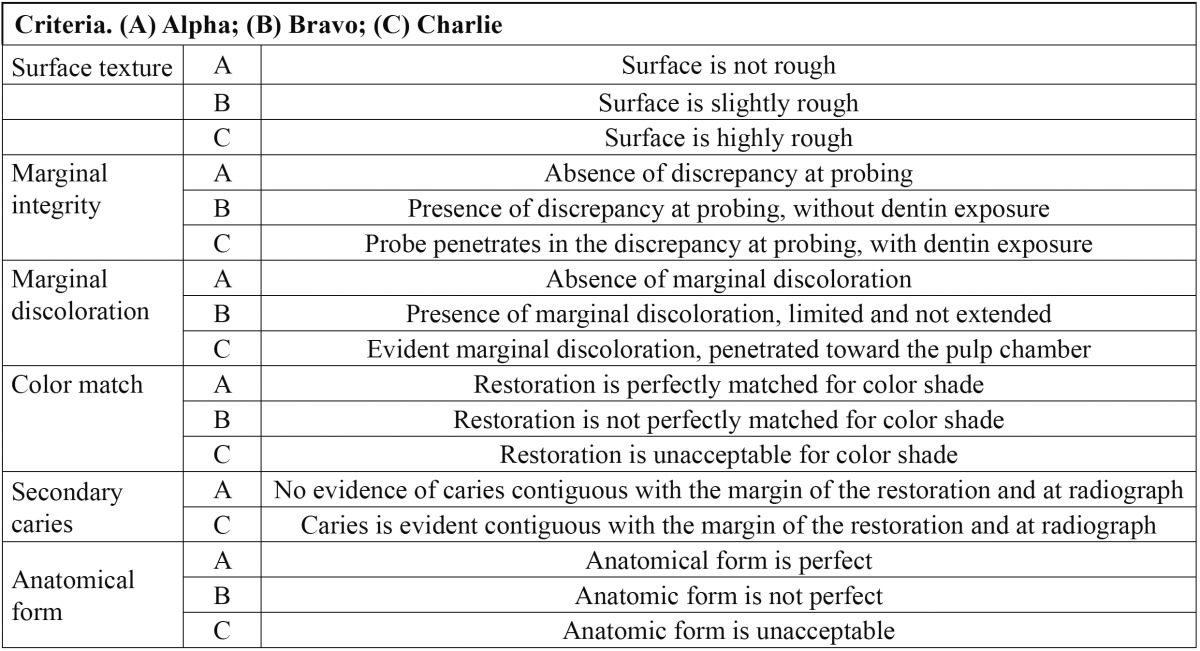
Modified USPHS Criteria.

-Statistical Analysis

Statistical evaluation was performed with program solution SPSS 17 [SPSS Inc, Chicago, IL, USA]. Since this is ordinal data structure, non-parametrical tests were used. The Mann-Whitney test was used to examine statistical differences between the two different materials used for cementation of the indirect restoration, according to the modified USPHS criteria. The Wilcoxon test was used to individually examine the difference between the results of the baseline and 10 years evaluation for each criterion. The standard value considered to demonstrate statistically significant differences was set at *p* <0.05.

## Results

All the patients attended the recall examinations. None of the restorations required replacement during or after 10 years of function.

The distribution of the evaluated restorations are summarized in  Table 3.

**Table 3:**
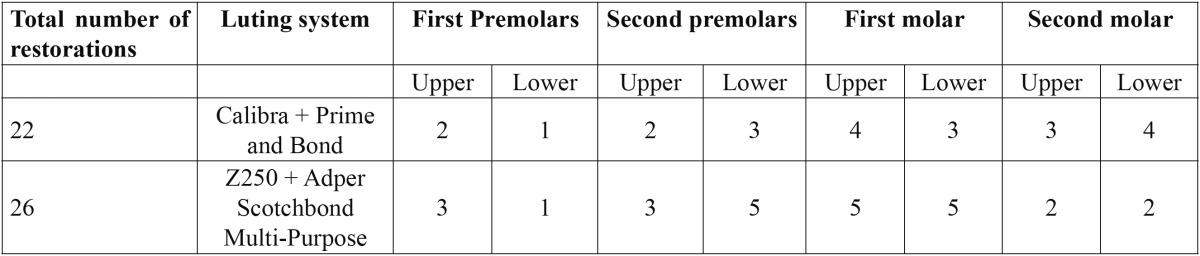
Number (n) and distribution of evaluated composite resin inlays/onlays.

Ratio women to men 15/8; with a mean age 37 years standard deviation 9 years, range of years 18-60.

Results of the clinical parameters comparing Group A and B inlays at baseline and at 10-years follow-up appointments are reported in table 4. Numerical but not statistically significant differences were noted on any of the recorded clinical parameters [*p*>0.05] between the inlay/onlays of Group A and Group B [first null hypothesis accepted].

Among the clinical parameters, after ten years of function, the anatomical form of the complete surface was scored as Alpha in 99% of Group A and 81% of Group B; marginal integrity was scored as Alpha in 45% of Group A and 54% of Group B; color match for Group A and B were scored respectively as Alpha in 73% and 81% of cases; marginal discoloration for Group A and B were scored respectively as Alpha in 41% and 46% of restorations; the surface texture for 65% of Group A was scored as Alpha while for group B was 84%. The clinical parameter secondary caries obtained 100% Alpha ratings for both groups. 9% and 7 % of Group A and B respectively received a Charlie rating for marginal discoloration criteria. Marginal integrity was scored as Charlie in 4% of Group A and 6% in Group B. Data are summerized in  Table 4.

**Table 4:**
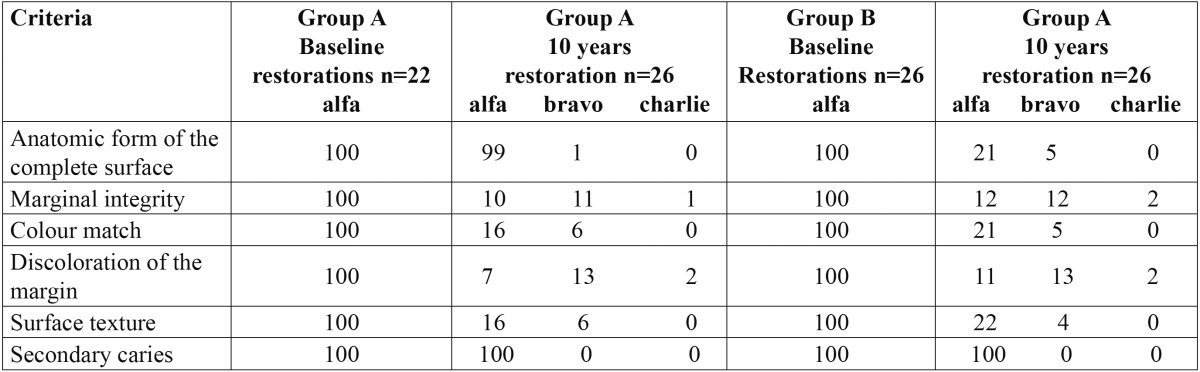
Indirect composite resin inlays: results of the clinical evaluation (modified USPHS scores, %) at baseline and 10-year follow-up (A = alfa, B = bravo, C = Charlie, D = delta). No statistically significant differences were noted on any of the recorded clinical criteria (*p* > 0.05) between the inlay/onlays of Group A and Group B at baseline and after ten years of function.

91% and 94 % of Group A and B respectively were rated as clinically acceptable in all the evaluated criteria ten years after clinical function [null hypothesis accepted].

## Discussion

In the present study, the indirect composite restorations showed after ten years of clinical function a high success rate of approximately 90%. The success rate found in the present study is comparable to other reports showing a succes rate for indirect composite inlays/onlays of 97.5% after 5 years of function (13) and 93 % after 2-3 years (21,22). In this clinical trial the restorations were carried out by an excellent clinician under optimal conditions and placed on patients specifically selected for good compliance. This could explain the high success value reported in this study.

The recall rate in this study was 100%. We expected a high compliance because the majority of the subjects in this study were young adult patients very concerned about their oral health.

In the present study two different luting methods were compared for bonding indirect restorations: a light-curing composite resin and a dual resin cement. Numerical but not statistically significant differences were noted on any of the recorded clinical parameters between the inlay/onlays of light cure or dual cure group. Therefore the first null hypothesis was accepted.

To our knowledge this is the first long term follow up trial comparing a light cure versus a dual cure luting pro-cedure for indirect composite inlays/onlays.

According to previous studies the probability of success of ceramic inlays placed with a light curing composite was only slightly lower than that of inlays placed with dual-curing composite (23-25).

The use of a light-curing composite resin for placing composite inlays has some practical advantages. As opposed to dual-curing composites, solely light-curing materials do not require two components to be mixed. This avoids an additional step in the work process and substantially lessens the danger of air bubbles getting into the luting composite. In addition, with the light-curing composite the clinician has plenty of time to remove excess material before polymerization starts. This is particularly important for operators with little experience, because excess composite that has hardened between teeth is very difficult to remove.

The most critical issue among posterior composite restorations is marginal discoloration and marginal integrity (26,27). Marginal discoloration is classified based on the penetration of dye into the pulp. In the present study two restoration of both groups showed evident marginal discoloration and were therefore rated as Charlie. Also for the parameter marginal integrity two restoration of both groups were recorded as failure because showed dentin exposure and were rated as Charlie. However 91% and 94 % of Group A and B respectively were rated as clinically acceptable showing rating as Alpha and Bravo in all the evaluated criteria ten years after clinical function [null hypothesis accepted]. Longevity of dental restorations is dependent upon many factors that are patient-, material and dentist-related (28).

In accordance with our results a meta-analysis on posterior restorations demonstrate annual failure rates for posterior composite resin inlays and onlays that range from 0%-10%, with a mean value of 2.9% [median, 2.3%] (27).

## Conclusions

Within the limits of the study the results showed after ten years of function a comparable clinical performance of indirect composite resin inlays/onlays placed with a light cure or dual cure luting procedures. Under controlled clinical conditions indirect composite resin inlays/onlays exhibited a succes rate of approximately 90% after ten years. The clinical performance of indirect composite resin restoration therefore is reliable for the restoration of large defects on a long term basis.
